# Outcomes of surgery for giant hepatic hemangioma

**DOI:** 10.1186/s12893-021-01185-4

**Published:** 2021-04-08

**Authors:** Qing-song Xie, Zi-xiang Chen, Yi-jun Zhao, Heng Gu, Xiao-ping Geng, Fu-bao Liu

**Affiliations:** Hepatopancreatobiliary Surgery, Department of general surgery, The First Affiliated Hospital of Anhui Medical University, 120# Wanshui Road, Hefei, 230022 Anhui China

**Keywords:** Hepatic hemangioma, Giant, Surgical management, Surgical indication, Surgical technique, 3D image reconstruction

## Abstract

**Background:**

The surgical indications for liver hemangioma remain unclear.

**Methods:**

Data from 152 patients with hepatic hemangioma who underwent hepatectomy between 2004 and 2019 were retrospectively reviewed. We analyzed characteristics including tumor size, surgical parameters, and variables associated with Kasabach–Merritt syndrome and compared the outcomes of laparoscopic and open hepatectomy. Here, we describe surgical techniques for giant hepatic hemangioma and report on two meaningful cases.

**Results:**

Most (63.8%) patients with hepatic hemangioma were asymptomatic. Most (86.4%) tumors from patients with Kasabach–Merritt syndrome were larger than 15 cm. Enucleation (30.9%), sectionectomy (28.9%), hemihepatectomy (25.7%), and the removal of more than half of the liver (14.5%) were performed through open (87.5%) and laparoscopic (12.5%) approaches. Laparoscopic hepatectomy is associated with an operative time, estimated blood loss, and major morbidity and mortality rate similar to those of open hepatectomy, but a shorter length of stay. 3D image reconstruction is an alternative for diagnosis and surgical planning for partial hepatectomy.

**Conclusion:**

The main indication for surgery is giant (> 10 cm) liver hemangioma, with or without symptoms. Laparoscopic hepatectomy was an effective option for hepatic hemangioma treatment. For extremely giant hemangiomas, 3D image reconstruction was indispensable. Hepatectomy should be performed by experienced hepatic surgeons.

## Introduction

Cavernous hemangioma, which is usually solitary and asymptomatic, is the most common benign tumor of the liver, with a reported prevalence of 3–20% based on autopsy series [1]. Hepatic hemangioma frequently presents in the fourth and fifth decades of life, and the female:male incidence ratio is 6:1. Giant hepatic hemangioma, which is defined as a tumor larger than 10 cm in diameter, is uncommon and rarely causes symptoms [2]. Giant hepatic hemangioma is present in less than 10% of cases [3].

Treatments for hepatic hemangioma include radiofrequency ablation (RFA), monoclonal antibody therapy, radiation therapy (RT), trans-arterial embolization (TAE), interferon therapy, liver transplantation, and surgical procedures (enucleation and resection). The therapeutic mechanism of RFA may be its induction of localized thermal injury on the flat endothelial cells constituting the walls of widely dilated non-anastomotic vascular spaces [4–6]. The advantages of RFA for the treatment of hepatic hemangioma include the lack of requirement for a safety margin and the ability to achieve clear shrinkage of the tumor around the ablation zone [7]. The main disadvantage of RFA for hepatic hemangioma is the risk of hemolysis, including hemoglobinuria, hemolytic jaundice, anemia, and even renal damage, esophageal perforation, and thrombosis. The risks of these adverse outcomes increase with tumor size [6, 8, 9]. The mechanism for the efficacy of RT may be its destruction of vascular endothelial cells and smooth muscle cells, resulting in vascular thrombosis, necrosis, and fibrosis [4, 10, 11]. However, because of treatment-related liver toxicity and the long-term potential for secondary malignancies, RT is rarely recommended as a first-line therapy for liver hemangioma [11]. TAE causes arterial blood supply blockage and subsequent shrinkage of tumors, with the potential complications of ischemia, intracavitary bleeding, and infection [12]. TAE is recommended to reduce hemangioma size, especially when the tumor is larger than 20 cm in diameter [13–16], and is used in cases of preoperative hepatic hemangioma rupture [17]. Liver transplantation due to giant hepatic hemangioma has been reported in patients with Kasabach–Merritt syndrome [18–22]; however, further research in this area is needed. Hepatic resection has been used to treat hepatic hemangioma since 1898 [23]. Approximately one century later, enucleation was first used for the treatment of hepatic hemangioma, with the reported advantages of safety, minimal blood loss, lower complication rates, and maximum preservation of the normal liver parenchyma [24–27]. However, as hepatic hemangiomas grow slowly and rarely rupture [28], surgical treatment is not always necessary. In addition, a study of 5542 cases of liver resection performed in the United States between 2005 and 2011 showed that the mortality rates at 30 postoperative days were 0.9% for 1164 cases with benign liver tumors and 1.4% for 4378 cases with metastatic liver cancer. The mortality rates were associated with age and surgical complications, but not with the nature of the lesions or the range of liver resection [29]. Thus, surgical treatment should be used with care for benign liver lesions. The present study was a retrospective analysis of surgical outcomes in patients with giant hepatic hemangiomas.

## Methods

### Patient selection

Between 2004 and 2019, 152 patients with the primary diagnosis of hepatic hemangioma were referred for surgical evaluation at the First Affiliated Hospital of Anhui Medical University. Hepatic hemangioma was diagnosed based on imaging studies, clinical data, and histological analyses. The indication for operation was giant (> 10 cm) liver hemangioma, with or without symptoms and Kasabach–Merritt syndrome (characterizing with thrombocytopenia and disseminated intravascular coagulation, and occasional fatal hemorrhage). Patients’ medical records were reviewed retrospectively and we report two notable cases.

### Statistical analysis

Results are presented as means ± standard errors of the mean. Analysis of variance, Student’s *t* test, and the *χ*^2^ test were used to determine the significance of differences between experimental groups. All statistical analyses were performed using SPSS software (ver. 19.0 for Windows; SPSS Inc., Chicago, IL, USA). *P* values < 0.05 were taken to indicate statistical significance.

## Results

The mean age of patients was 51.6 (range, 26–71) years, and most (61.8%) patients were female. Most (63.8%) patients with hepatic hemangioma were asymptomatic, and 29.6% of patients had hepatic hemangiomas larger than 15 cm (Table 1). Among 22 patients with Kasabach–Merritt syndrome, 86.4% of hepatic hemangiomas were larger than 15 cm. Most (54.5%) patients with Kasabach–Merritt syndrome had abdominal pain. The number of platelets in patients (range, 56 × 10^9^/l–89 × 10^9^/l) increased after surgery (range, 75 × 10^9^/l–143 × 10^9^/l) (Table 2).

**Table 1:** Background characteristics of patients undergoing hepatectomy for hemangioma

Basic data	N
Number of cases	152
Age (years)	
Mean ± SD	51.6 ± 9.6
Range	26–71
Gender	
Male (%)	58 (38.2)
Female (%)	94 (61.8)
Symptoms	
No symptoms (%)	97 (63.8)
Abdominal pain (%)	55 (36.2)
Tumor size	
> 10 cm and < 15 cm (%)	107 (70.4)
> 15 cm (%)	48 (29.6)

**Table 2:** Background characteristics of patients with Kasabach–Merritt syndrome undergoing hepatectomy for hemangioma

Basic data	N
Number of cases	22
Age (years)	
Mean ± SD	56.4 ± 10.6
Range	36–71
Gender	
Male (%)	7 (31.8)
Female (%)	15 (68.2)
Symptoms	
No symptoms (%)	10 (45.5)
Abdominal pain (%)	12 (54.5)
Platelet count (× 10^9^/l)	
Pre-operation (range)	56–89
Post-operation (range)	75–143
Tumor size	
> 10 cm and < 15 cm (%)	3 (13.6)
> 15 cm (%)	19 (86.4)

Most (64.5%) hemangiomas were solitary. Hepatic hemangiomas were treated by enucleation (30.9%), sectionectomy (28.9%), hemihepatectomy (25.7%), and the removal of more than half of the liver (14.5%) through open (87.5%) and laparoscopic (12.5%) approaches (Table 3). Compared with open hepatectomy, laparoscopic hepatectomy was associated with a similar operative time, estimated blood loss, and major morbidity and mortality, but a shorter length of stay (*P* < 0.01) (Table 4).

**Table 3:** Surgical parameters of patients who underwent hepatectomy for hemangioma

Variables	Data
Tumor number	
Solitary (%)	98 (64.5)
Multiple (%)	54 (35.5)
Tumor size (cm)	
Mean ± SD	12.9 ± 3.3
Range	10–23
Hepatectomy procedure	
Enucleation (%)	47 (30.9)
Sectionectomy (%)	44 (28.9)
Hemihepatectomy (%)	39 (25.7)
More than hemihepatectomy (%)	22 (14.5)
Operational approach	
Open approach	133 (87.5)
Laparoscopic approach	19 (12.5)
Operative time (min) (range)	177 (60–300)
Estimated blood loss (ml) (range)	343 (10–1200)
Major morbidity or mortality	0

**Table 4:** Surgical parameters of patients who underwent hepatectomy for hemangioma

Variables	Open approach	Laparoscopic approach	*P*
N (%)	133 (87.5)	19 (12.5)	
Age (mean ± SD)	52.8 ± 9.4	50.8 ± 11.9	0.400
Gender			
Male	50 (37.6)	11 (57.9)	0.091
Female	83 (62.4)	8 (42.1)	
Tumor size (cm) (mean ± SD)	13.0 ± 3.4	12.0 ± 2.6	0.152
Operative time (min) (mean ± SD)	167.4 + 57	177.6 ± 59.4	0.488
Estimated blood loss (ml) (mean ± SD)	319 ± 245	282 ± 190	0.537
Major morbidity or mortality	0	0	
LOS (mean ± SD)	8 ± 0.8	5 ± 0.6	< 0.01*

*LOS* Length of stay

*Significant difference

**Fig. 1:**
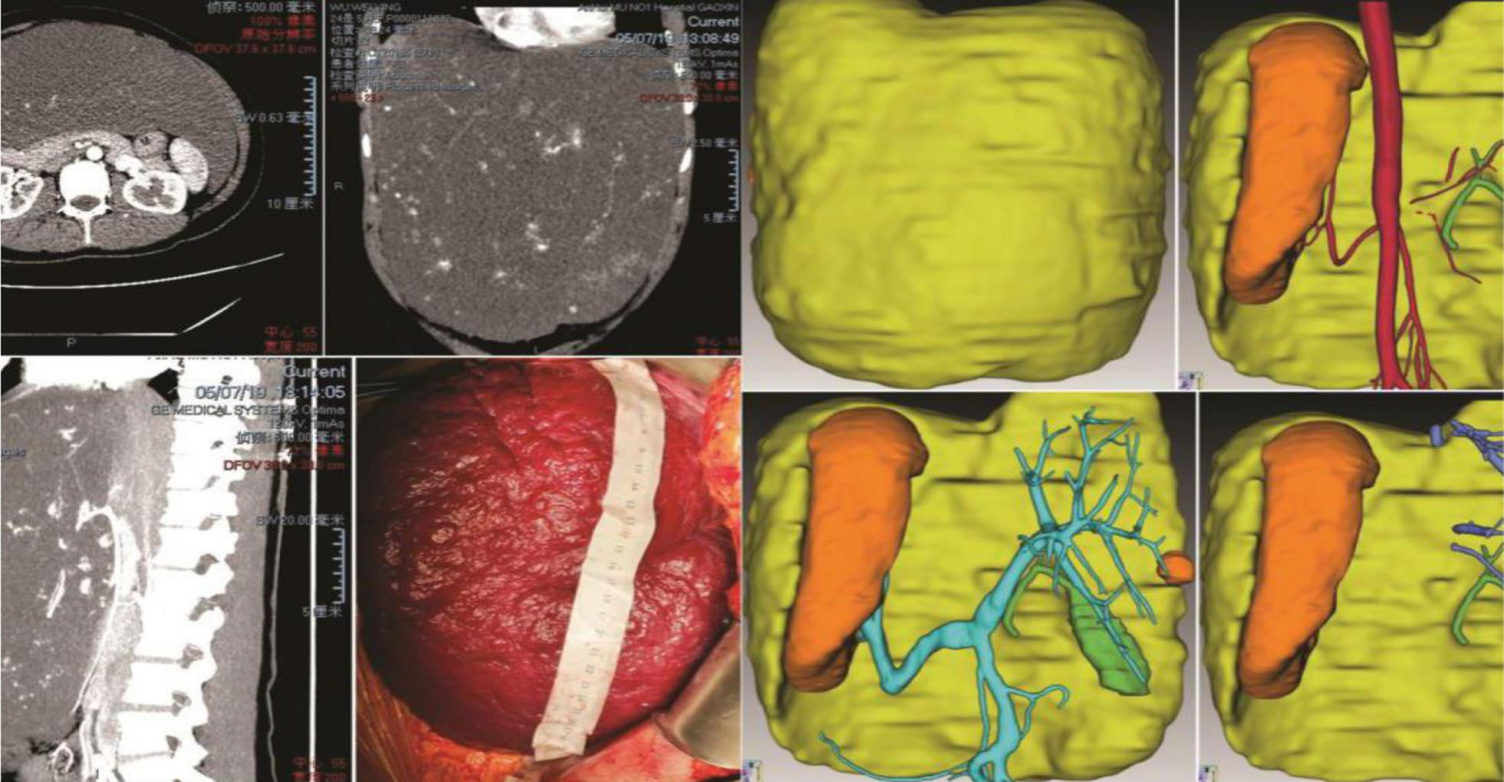
A 24-year-old woman with two children was diagnosed with an extremely giant hepatic hemangioma in the left lobe of her liver after reporting abdominal pain. The tumor, extending below the belly button, had a maximum diameter of 38 cm. The 3D-reconstructed image shows the tumor (yellow), normal liver (orange), and the relationships between the tumor and hepatic vessels. The patient also had Kasabach–Merritt syndrome. The number of platelets increased from 57 × 10^9^/l to 100.5 × 10^9^/l after surgery

For patients with complex and especially large hepatic hemangiomas, three-dimensional (3D) image reconstruction was performed to understand future remnant liver volumes and the relationships between intrahepatic blood vessels and tumors (Fig. 1).

**Fig. 2:**
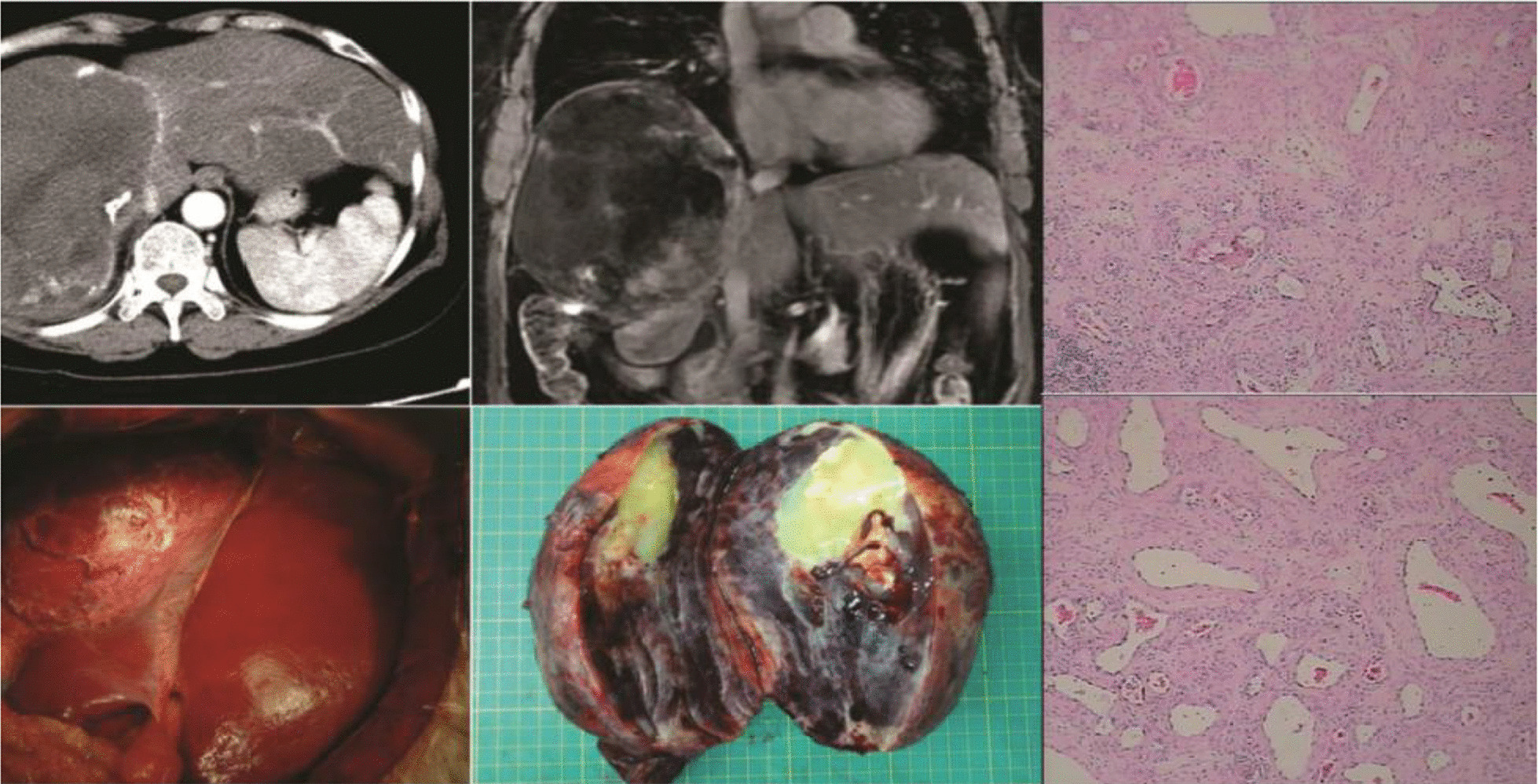
A 62-year-old female patient with a giant hemangioma in the right lobe of the liver was misdiagnosed with hepatocellular carcinoma. In 2006, the patient underwent physical examination, which revealed a liver mass. The patient was diagnosed with hepatocellular carcinoma and underwent five rounds of transhepatic arterial chemotherapy and embolization and multiple cytokine-induced killer cell treatments at another hospital. In December 2015, the patient was admitted to our hospital and we determined that she was not infected with hepatitis b virus and was α-fetoprotein negative. The CT image shows a large mass in the right lobe of the liver. The patient’s liver reserve function was normal. She underwent extensive right hepatectomy and was given a final pathological diagnosis of hepatic hemangioma

One notable case was that a 62-year-old female patient with a giant hemangioma in the right lobe of the liver was misdiagnosed with hepatocellular carcinoma. In 2006, the patient’s physical examination revealed a liver mass. The patient was diagnosed with hepatocellular carcinoma and underwent five rounds of transhepatic arterial chemotherapy and embolization and multiple cytokine-induced killer cell treatments at another hospital. In December 2015, the patient was admitted to our hospital and we determined that she was not infected with hepatitis B virus and was alpha-fetoprotein negative. Computed tomography (CT) revealed a large mass in the right lobe of the liver, and the patient’s liver reserve function was normal. Thus, she underwent extensive right hepatectomy, and the final pathologically confirmed diagnosis was hepatic hemangioma (Table 5; Fig. 2).

**Table 5:** One case of giant hemangioma in the right lobe of the liver misdiagnosed as hepatocellular carcinoma

Variables	Data
Age	62 years old
Gender	Female
BMI	27.48 kg/m
Symptoms	Abdominal pain
Laboratory data	HBV(−), AFP(−), ICG15 min (2.8%), Child–Pugh (A)
Tumor location and size	Right lobe of liver, D = 17 cm and weighted about 1500 g
Past medical history	5 rounds of TACE, multiple CIK treatments
Hepatectomy procedure	Extensive right hepatectomy
Operation and portal occlusion time	120 and 20 min
Estimated blood loss	170 ml
Pathological findings	Hepatic hemangioma

*HBV* Hepatitis b virus, *AFP* alpha fetoprotein, *ICG* 15-min indocyanine green clearance, *TACE* Transhepatic arterial chemotherapy and embolization, *CIK* Cytokine-induced killer cell

**Fig. 3:**
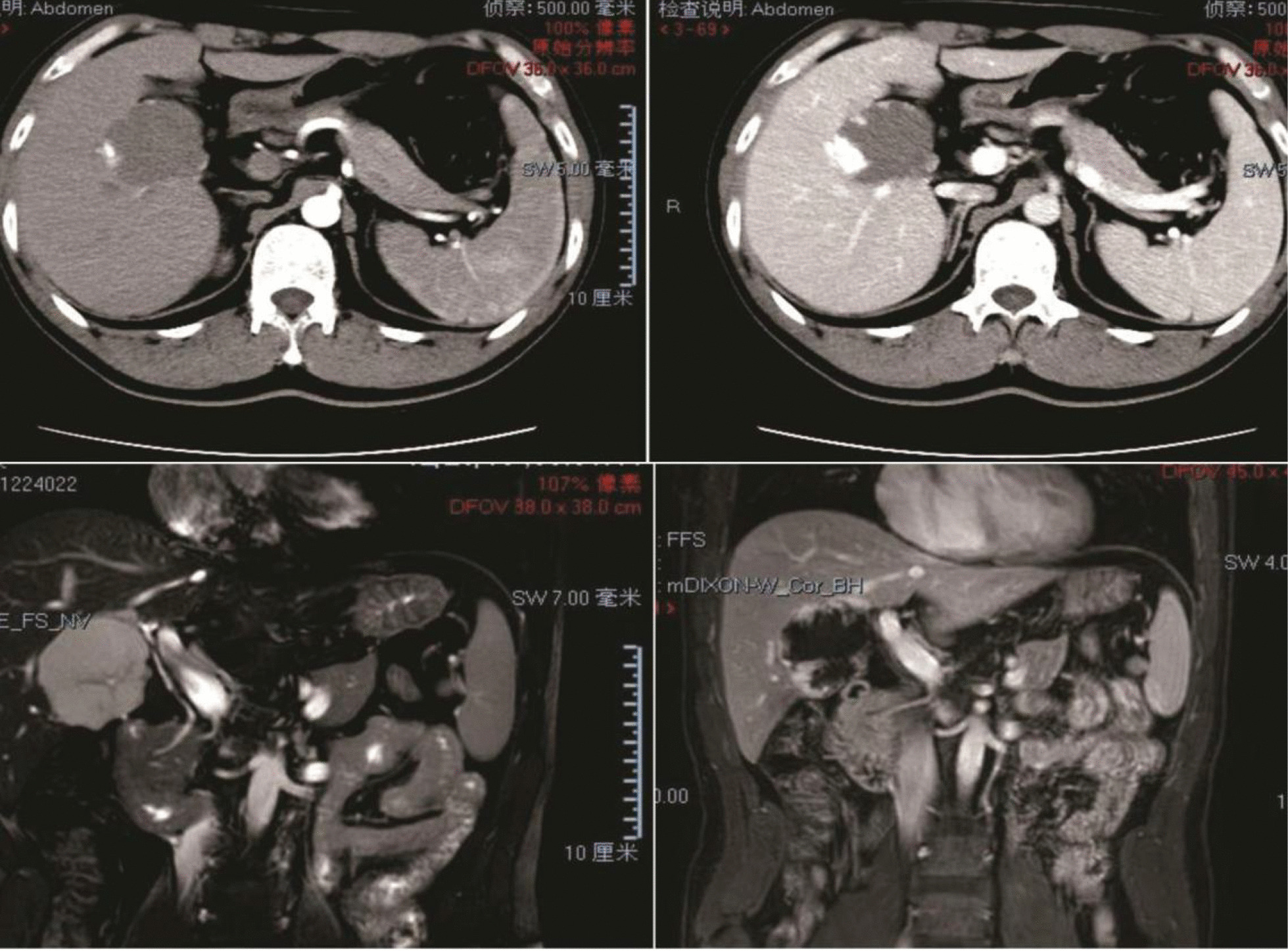
A 45-year-old male patient, a seaman, had symptoms of obstructive jaundice and was diagnosed with a hepatic hemangioma about 5 cm in diameter near the first porta hepatis. CT and MRI images show the hepatic hemangioma at the first porta hepatis, compressing the intrahepatic bile duct, which caused obstructive jaundice. We enucleated the tumor and the patient’s bilirubin level returned to normal

Another significant case occurred in a 45-year-old male patient (a seaman) with symptoms of obstructive jaundice who had a ~ 5-cm hepatic hemangioma near the first porta hepatis. CT and magnetic resonance imaging (MRI) revealed that the hepatic hemangioma was located at the first porta hepatis and compressed the intrahepatic bile duct, resulting in obstructive jaundice. We enucleated the tumor and the patient’s bilirubin level returned to normal (Table 6; Fig. 3).

**Table 6:** One case of giant hepatic hemangioma near the first porta hepatis

Variables	Data
Age	45 years old
Gender	Male
BMI	22.2 kg/m^2^
Symptom	Obstructive jaundice
Total bilirubin (TBIL)	Pre/post-operation: 123.5/18.7 μmol/l
Direct bilirubin (DBIL)	Pre/post-operation: 100.8/10.3 μmol/l
Indirect bilirubin (IBIL)	Pre/post-operation: 22.7/8.4 μmol/l
Laboratory data	HBV(−), AFP(−)
Tumor location and size	Near the first porta hepatis and 5.8 cm
Past medical history	None
Hepatectomy procedure	Enucleation
Operation and portal occlusion time	65 and 0 min
Estimated blood loss	100 ml

*HBV* Hepatitis b virus, *AFP* alpha fetoprotein

**Fig. 4:**
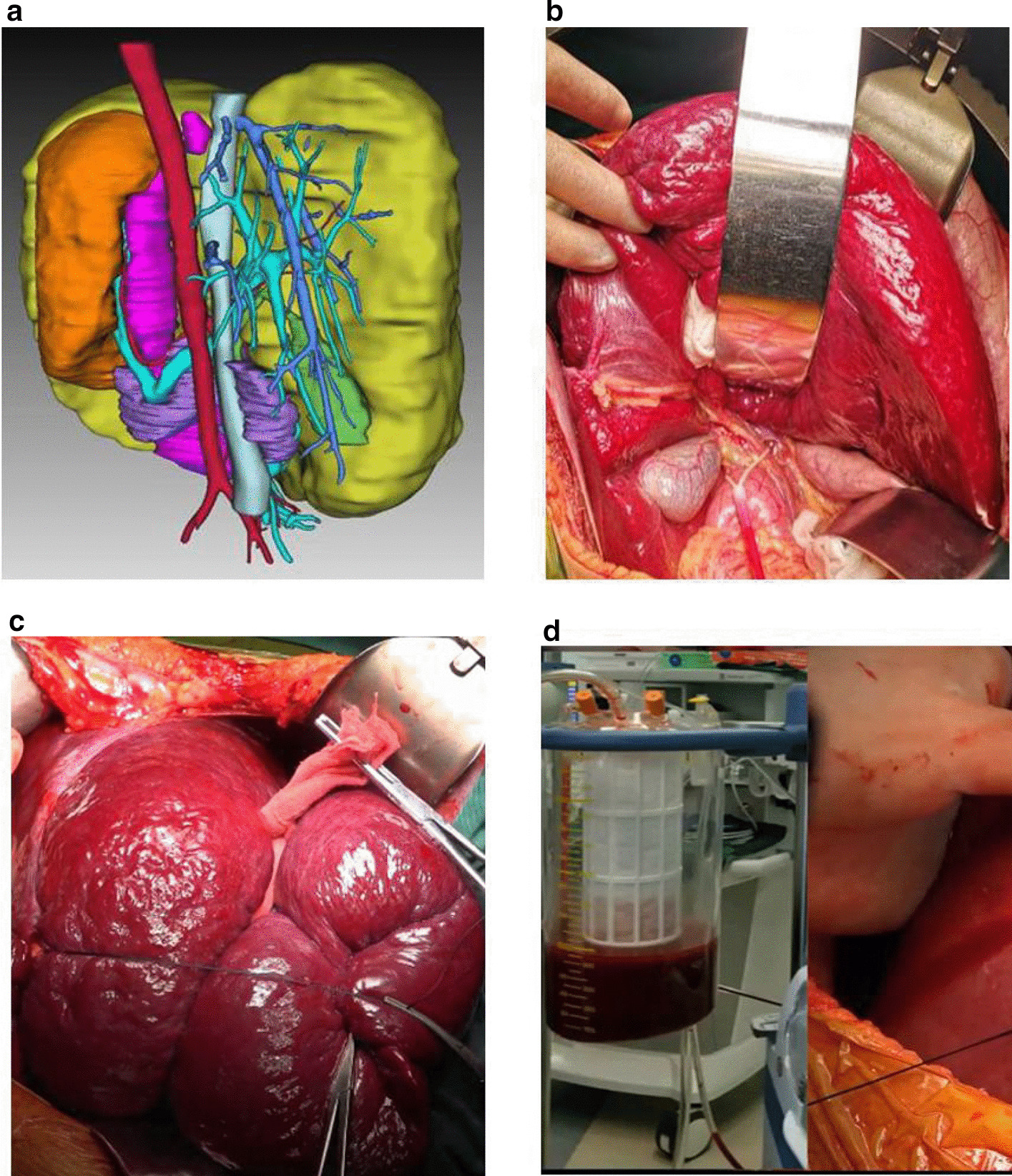
Methods for the prevention of intraoperative bleeding. **a** 3D image reconstruction; **b** Prearrangement of a hepatic portal blocking band; **c** Bandaging of the tumor; **d** Autologous blood transfusion

For extremely giant hemangiomas, the surgical procedure was as follows. First, 3D image reconstruction was performed and radiographic results were studied scrupulously to design an optimal operative protocol. Next, a transverse incision was made in the epigastrium corresponding to the longest diameter of the tumor to fully expose the tumor and first porta hepatis. For obese patients or especially giant tumors, incisions extended to the xiphoid process. The first porta hepatis was exposed and the hepatic portal blocking band was prearranged; then, the hepatic artery and portal vein on the tumor side were ligatured and severed to shrink the tumor. The second porta hepatis was exposed and dissected. The tumor was bandaged to reduce bleeding, and liver resection was performed using hectogram pliers along the border between the liver and tumor. The intrahepatic small vessels and bile ducts were disposed of using titanium clips. Autologous blood transfusion was performed, and the middle hepatic vein was carefully protected as much as possible. Finally, the hepatic cutting surface was sutured with 4-0 prolene for hemostasis, and drainage tubes were placed under the liver and on the liver section (Fig. 4).

## Discussion

Hepatic hemangioma is the most common benign liver tumor [30–32]. Some hemangiomas contain estrogen receptors and grow during pregnancy, oral contraceptive use, or Rogaine^®^ and/or steroid administration [4, 33, 34]. Thus, female sex hormones play an important role in the development of hepatic hemangioma, and these tumors are more common in women. Nearly two-thirds (61.8%) of patients with hepatic hemangioma in our sample were female, consistent with previously reported findings [4, 34].

As the treatment strategy for hepatic hemangioma depends on tumor size, the definition of giant hepatic hemangioma is crucial. Different criteria for this definition have been reported. The majority of authors use the criterion of diameter > 4 cm [2, 35], whereas others use diameter > 5 cm [36] and a minority of authors use diameter > 10 cm [37]. We believe that the definition of giant hemangioma should be based on diameter ≥ 10 cm, as 10-cm hemangiomas can cause more serious symptoms than can 4-cm tumors. Thus, we focused on patients with hepatic hemangiomas larger than 10 cm in this study.

The case of the female patient with a hepatic hemangioma misdiagnosed as hepatocellular carcinoma clarifies the importance of the accurate diagnosis of hepatic hemangioma. The diagnostic methods for hepatic hemangioma include ultrasonography, CT, MRI, scintigraphy and positron-emission tomography combined with CT, angiography, and biopsy. The central non-enhancing components, central hemorrhagic changes, and intralesional arterio-portal or arterio-venous shunts of giant (> 10 cm) hemangiomas may lead to misdiagnosis [37–39]. Thus, the differentiation of hemangioma from liver metastasis in patients with primary liver neoplasms and from hepatocellular carcinoma in patients with cirrhosis is important. 3D visualization is an alternative for diagnosis and surgical planning for partial hepatectomy [40–43]. In our sample, we evaluated hepatic hemangiomas routinely using B-mode ultrasonography, MRI, CT, and serum tumor markers. Imaging consultation was adopted for intractable cases. We also used 3D reconstruction for further diagnosis and surgical planning in cases of complex and giant hepatic hemangiomas.

Hepatic hemangiomas tend to grow slowly and rupture infrequently [28, 44]. As patients with hepatic hemangioma can be considered to be ‘normal,’ surgical indications and techniques must be strictly controlled. Japanese surgeons have deemed that surgical resection may be justified for tumors less than 5 cm in diameter when malignancy is suspected; that patients with abdominal symptoms or coagulopathy are candidates for surgery when the tumor diameter exceeds 10 cm; that hepatectomy should be performed by experienced hepatic surgeons for tumors larger than 15 cm; and that asymptomatic hepatic hemangiomas (5–10 cm) require no surgical intervention [45]. In the current study, most tumors were larger than 10 cm, of which 29.6% were larger than 15 cm. About one-third (36.2%) of patients had symptoms, and 22 patients had Kasabach–Merritt syndrome. The sample included an exceptional case of a hemangioma less than 10 cm in diameter located at the first porta hepatis. As this small tumor resulted in obstructive jaundice and the patient was a seaman, we enucleated it.

Hepatic resection has historically been the most common therapy for hepatic hemangioma [23]. However, enucleation is now used more frequently and is favored by surgeons due to its safety and the reduction of blood loss and complication rates [24, 25, 27, 46]. The location of a tumor determines the surgical plan. Hepatectomy should be performed when the hepatic hemangioma is located deep within the liver parenchyma, does not present a surface free from the Glisson capsule, or occupies the entire lobe [47]. In the present study, the majority (69.1%) of patients underwent hepatectomy, and 14.5% of patients underwent extended hemihepatic resection. Only 30.9% of patients underwent enucleation, as nearly all tumors were more than 10 cm in diameter; 29.6% of tumors were greater than 15 cm in diameter, frequently occupying more than half of the liver.

Laparoscopic liver resection is used widely for hepatic hemangioma because it decreases postoperative complications, enables more rapid patient discharge, and supports better postoperative cosmetic satisfaction [48–52]. Laparoscopic resection of even an extremely giant (> 20 cm) hepatic hemangioma has been reported [53]. However, the results of randomized clinical trials comparing laparoscopic to open liver resection are not yet available, and the learning curve for laparoscopic resection and scoring systems for the degree of its difficulty will continue to evolve [54]. The results of a recent non-randomized controlled trial suggest that a laparoscopic approach to hepatic hemangioma treatment improves short-term surgical outcomes [55]. Our data revealed no difference in the operative time, estimated blood loss, and major morbidity and mortality between laparoscopic and open liver resection for hepatic hemangioma, with a significant reduction in the length of stay associated with laparoscopic liver resection. Although our study was retrospective, we recommend laparoscopic hepatectomy as a treatment option for hepatic hemangioma.

Kasabach–Merritt syndrome is characterized by the occurrence of disseminated intravascular coagulation due to hepatic hemangioma, and has a fatality rate of 7–10% that rises to 80% in the first year [56]. Hepatectomy [56], liver transplantation [18, 22], and steroid or beta-blocker therapy [57] have been reported to effectively treat Kasabach–Merritt syndrome in patients with hepatic hemangioma. Many patients with Kasabach–Merritt syndrome in our sample had extremely giant (> 15 cm) tumors, which made surgery more difficult.

We summarize our center’s surgical recommendations for giant hepatic hemangioma as follows. A large curved incision should be created in the upper abdomen to expose the tumor body and the first porta. The first hilum of the liver should be exposed as much as possible, the hepatic artery/portal vein of the affected side should be ligated and devascularized to shrink the tumor, and the liver parenchyma should be transected as far as possible within 30 min after blocking while avoiding repeated opening to minimize blood loss. Hemihepatic blood flow occlusion, hepatic inflow occlusion, and complete hepatic vascular occlusion should be applied, and highly efficient electrosurgical instruments should be used to resect the liver. Intratumoral blood transfusion should be performed and a low central venous pressure should be maintained during the operation. Multiple small tumors should not be treated simultaneously, and TAE should not be used unless the tumor ruptures; radiofrequency or microwave treatment should not be used at all.

## Conclusion

The present study showed that the indication for operation was giant (> 10 cm) liver hemangioma, with or without symptoms, and that laparoscopic hepatectomy was an effective treatment option for hepatic hemangioma. For extremely giant hemangiomas, 3D image reconstruction was indispensable. Hepatectomy should be performed by experienced hepatic surgeons.

## Data Availability

All data generated or analysed during this study are available from the corresponding author on reasonable request.

## Abbreviations

RFA: Radiofrequency ablation
RT: Radiation therapy
TAE: Trans-arterial embolization
3D: Three-dimensional
CT: Computed tomography
MRI: Magnetic resonance imaging
LOS: Length of stay
HBV: Hepatitis b virus
AFP: Alpha fetoprotein
ICG: 15-Min indocyanine green clearance
TACE: Transhepatic arterial chemotherapy and embolization
CIK: Cytokine-induced killer cell
